# Structure and properties of transcriptional networks driving selenite stress response in yeasts

**DOI:** 10.1186/1471-2164-9-333

**Published:** 2008-07-15

**Authors:** Hélène Salin, Vivienne Fardeau, Eugenia Piccini, Gaelle Lelandais, Véronique Tanty, Sophie Lemoine, Claude Jacq, Frédéric Devaux

**Affiliations:** 1 Laboratoire de génétique moléculaire, ENS/CNRS UMR 8541 46 rue d'Ulm, 75005 Paris, France; 2 Plate-forme transcriptome, IFR 36, 46 rue d'Ulm, 75005 Paris, France; 3 Muséum national d'Histoire naturelle, 57 rue Cuvier 75005 PARIS, France; 4 Commissariat à l'Energie Atomique, Institut de Biologie et de Technologies de Saclay, 91191 Gif sur Yvette Cedex, France; 5 Equipe de Bioinformatique Génomique et Moléculaire, INSERM UMR S726, Université Paris 7, 2 place Jussieu, 75251 Paris cedex 05, France

## Abstract

**Background:**

Stress responses provide valuable models for deciphering the transcriptional networks controlling the adaptation of the cell to its environment. We analyzed the transcriptome response of yeast to toxic concentrations of selenite. We used gene network mapping tools to identify functional pathways and transcription factors involved in this response. We then used chromatin immunoprecipitation and knock-out experiments to investigate the role of some of these regulators and the regulatory connections between them.

**Results:**

Selenite rapidly activates a battery of transcriptional circuits, including iron deprivation, oxidative stress and protein degradation responses. The mRNA levels of several transcriptional regulators are themselves regulated. We demonstrate the existence of a positive transcriptional loop connecting the regulator of proteasome expression, Rpn4p, to the pleiotropic drug response factor, Pdr1p. We also provide evidence for the involvement of this regulatory module in the oxidative stress response controlled by the Yap1p transcription factor and its conservation in the pathogenic yeast *C. glabrata*. In addition, we show that the drug resistance regulator gene *YRR1 *and the iron homeostasis regulator gene *AFT2 *are both directly regulated by Yap1p.

**Conclusion:**

This work depicted a highly interconnected and complex transcriptional network involved in the adaptation of yeast genome expression to the presence of selenite in its chemical environment. It revealed the transcriptional regulation of *PDR1 *by Rpn4p, proposed a new role for the pleiotropic drug resistance network in stress response and demonstrated a direct regulatory connection between oxidative stress response and iron homeostasis.

## Background

The adaptation of genome expression to the chemical environment is a complex but crucial challenge for all living cells. Functional genomics analyses in budding yeast have shown that environmental stress responses may involve rapid changes in the expression of up to 30% of the genome. A common response to all stresses, named ESR (Environmental Stress Response), has been described, which consists in the inhibition of the cytosolic translation apparatus and the activation of the energy storage pathways [1]. However, pathways responding specifically to the parameters of the environment also form a key part of the stress response. These pathways involve specific transcriptional modules that rapidly sense the environment as a series of chemical and physical features (e.g. redox, pH, osmolarity, temperature, etc.) and act together to adapt genome expression to the specific nature of each stress [2]. For instance, at least eight different transcription factors act together to define the first-hour response of yeast cells to the toxic metalloid arsenite [3]. These global and rapid responses are highly dynamic, involving sequential waves of gene activation and repression [1,2,4]. This requires tight temporal coordination between different transcriptional routes, which can be achieved in two complementary ways. First, the transcription factors involved in stress responses, despite responding to different signals, may have overlapping sets of targets [5]. Second, cross-regulation between transcription factors may ensure the coordinated activation of different pathways [6]. We focus here on the cross-talks between three transcriptional modules responsible for the oxidative stress response, the ubiquitine-mediated protein degradation and the pleiotropic drug resistance, respectively. These cellular pathways exist in all species, from bacteria to mammals and plants. In *S. cerevisiae*, the oxidative stress response is controlled principally by the Yap1p transcription factor of the AP1-like leucine zipper family. Yap1p acts as a secondary sensor for oxidative molecules, and thus responds to a wide spectra of toxic compounds, such as hydrogen peroxide, metals and metalloids, organic nucleophilic molecules and internal metabolic oxidative stress due to the production of toxic by-products during glycolysis [3,4,7,8]. Yap1p recognizes YRE (Yap1p response elements, 5'-TKACTMA-3') in the promoters of genes involved in redox homeostasis and in xenobiotic export at the plasma membrane. The proteasome is involved in both the degradation of damaged or aggregated proteins and in the post-translational regulation of several biological processes, playing a key role in many stress responses [9]. Expression of the genes involved in proteasome biogenesis and activity, and in ubiquitin-dependent proteolysis, is controlled by the C_2_H_2 _zinc finger protein Rpn4p, which recognizes the PACE (proteasome associated control element, 5'-GGTGGCAAA-3') sequence in the promoter of its target genes [3,10,11]. Pleiotropic drug resistance involves the upregulation of membrane proteins involved in drug efflux. The corresponding genes are controlled principally by two Zn_2_Cys_6 _Gal4p-like transcription factors: Pdr1p and Pdr3p. These two transcription factors have largely overlapping sets of targets and recognize the same DNA motif (named PDRE, 5'-TCCGYGGR-3'), but have different roles and regulatory properties [2,5,12-14]. The Yap1p and Rpn4p pathways are simultaneously involved in the yeast response to arsenate [3]. Yap1p acts together with the Pdr1p/Pdr3p pathway to induce a drug specific response to the antifungal drug benomyl [4]. No transcriptional regulation has been described for *PDR1*, but *YAP1*, *RPN4 *and *PDR3 *are induced by stress [3,5,6]. In this work, we used selenite as a model stress to investigate further the interactions between these three transcriptional modules. Selenium is an essential oligoelement that replaces the sulfur atom of some methionine and cysteine in proteins involved in various essential cell functions [15]. Selenium is also a promising agent for cancer therapy and anti-aging treatments [16]. However, high doses of selenium are toxic to eukaryotic cells [17]. In yeast, selenium alters genome stability [18] and is detoxified in the vacuole after reacting with glutathione [19]. Yeast cells have a high level of selenium tolerance, and yeast enriched in selenium have been used in therapeutic trials [20]. We showed in this study that toxic doses of selenite activated various yeast stress response pathways, including the proteasome, oxidative stress, iron homeostasis and general stress pathways. We demonstrated that, in these growth conditions, the expression of *PDR1 *and *RPN4 *was coordinated through a positive transcriptional loop. This loop contributed to the optimal Yap1p-dependent oxidative stress induction of several genes encoding membrane proteins, including *FLR1*, *ATR1 *and *FRM2*. This function seemed to be conserved in the pathogenic yeast species *C. glabrata*. Finally, our data provide evidence for direct transcriptional regulation of the iron homeostasis regulator Aft2p and of the multidrug resistance regulator Yrr1p by Yap1p, indicating a broader role for this factor in coordination of the oxidative stress response.

## Results

### Gene ontology mapping of the selenite response

**Figure 1:**
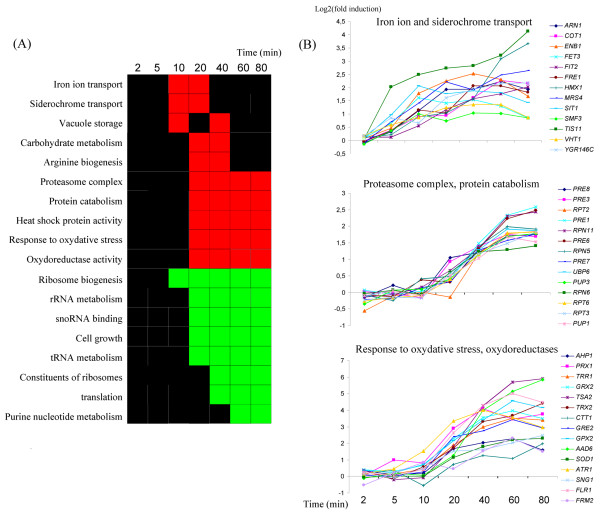
**Global Gene Ontology mapping of the selenite response in *S. cerevisiae***. (A): The gene ontology categories with levels of expression significantly changed by selenite were identified by T-profiler [22]. The graph represents only the most significant functional categories, as a function of time. More complete T-profiler results are available in additional file 3. The color code is as follows: black: E-value > 0.05 (non significant variation); red: E-value < 0.05 and t-value > 0 (positive significant variation); green: E-value < 0.05 and t-value < 0 (negative significant variation). (B): Gene expression patterns for the functional categories discussed further in the text. Wild-type cells were treated with selenite and gene expression was evaluated by microarray analyses at different time points, using untreated cells as a reference. Note that the lists of genes represented here are not exhaustive and represent a sample of the genes present in these GO categories. More complete information can be found in additional file 1.

**Figure 2:**
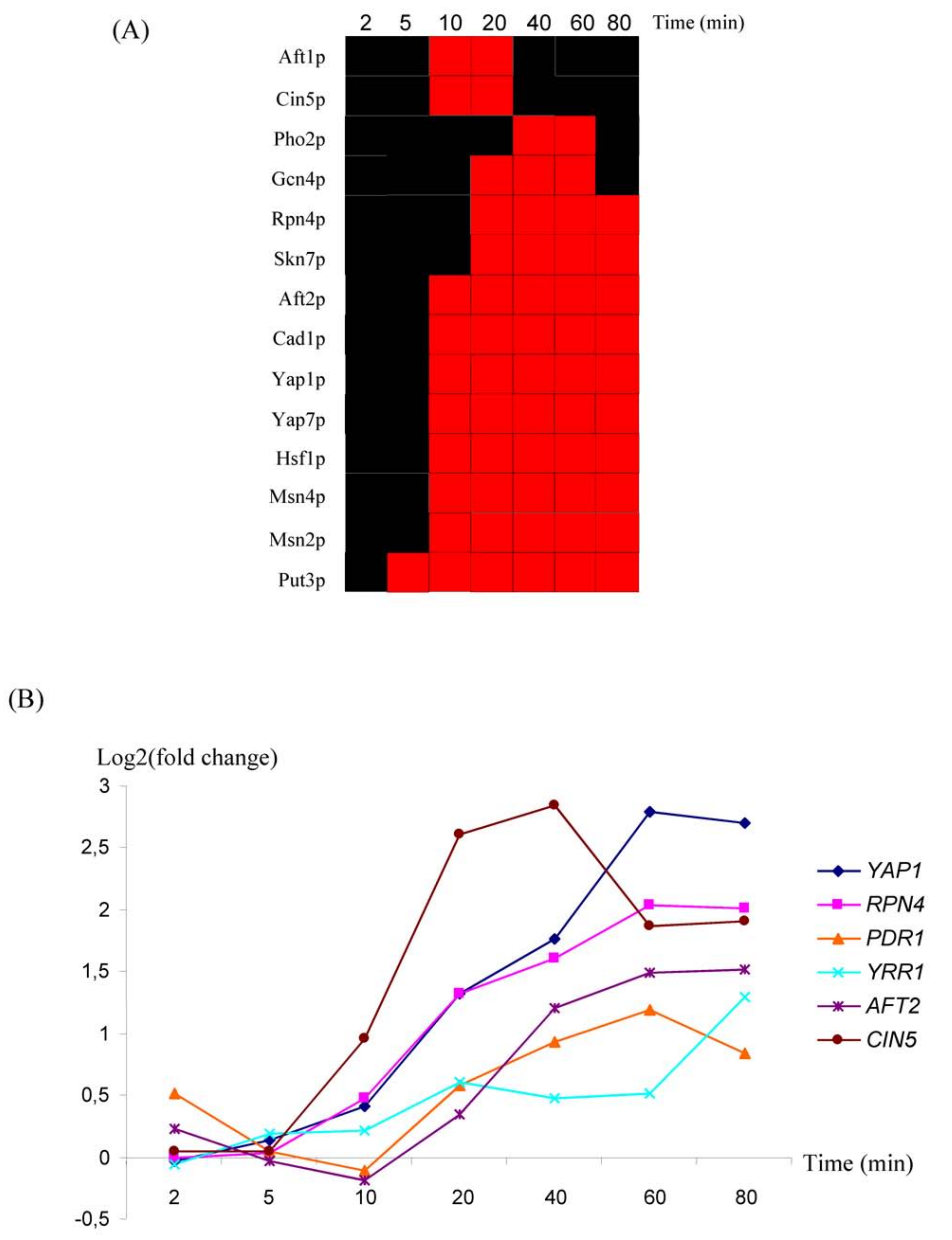
**Transcriptional regulation and selenite response**. (A): T-profiler prediction of the transcription factors involved in the positive response of yeast to selenite. The color code is the same as for figure 1A. These predictions were based on previous ChIP-chip results [30]. (B): Expression patterns of genes encoding transcription factors involved in stress response pathways. Full results are presented in additional file 1. Wild-type cells were treated with selenite and gene expression was evaluated by microarray analyses at different time points, using untreated cells as a reference.

We analyzed the transcriptome of *S. cerevisiae *cells treated with 1 mM sodium selenite for 2, 5, 10, 20, 40, 60 and 80 minutes. The corresponding cDNAs were competitively hybridized on DNA microarrays, with cDNAs obtained from cells mock-treated for an equivalent period. Each experiment was carried out four times, with independent biological samples. We used SAM to evaluate the significance of variations in expression of each gene [21]. The dose of selenite used was the lowest dose that significantly altered cell survival in our preliminary growth assays (data not shown). This toxic dose of selenite induced large changes in the transcriptome of the cells (see additional file 1). In our data set, about 30% of the yeast ORFs displayed a significant change in expression level for at least two consecutive time points, with similar numbers of up- and downregulated. The earliest significant effects on gene expression were detected at 10 minutes, with the peak response at 40 minutes (see additional file 2 and additional file 1). We investigated the functional pathways involved in this large and complex response using the t-profiler software [22], which identified the Gene Ontology (GO) categories significantly up- or downregulated over the whole dataset (figure 1A, see additional file 3). T-profiler analysis indicated that iron homeostasis genes were among the first to be induced (figure 1A). Selenite also induced an oxidative stress response characterized by the up-regulation of genes involved in redox homeostasis and in proteasome activity (figure 1A). Finally, our selenite treatment, similarly to all stresses with deleterious effects on growth, triggered a large ESR [1]. This response was characterized by the general repression of genes involved in cytosolic translation (ribosome biogenesis, rRNA and tRNA processing, etc.) and by the induction of chaperone proteins and genes involved in energy storage and carbohydrate metabolism (figure 1A). This probabilistic model of the selenite response was confirmed by the expression profiles of genes from the GO categories cited above (figure 1B and see additional file 4). We also used T-profiler to predict the transcription factors likely to be responsible for the gene expression patterns observed, based on previous genome-wide chromatin immunoprecipitation experiments and DNA motifs in the promoters of the corresponding genes (figure 2A). This led to the identification of 14 transcription factors as possibly positively regulating gene expression in response to selenite (figure 2A, see additional file 3). This list includes regulators involved in the iron homeostasis (Aft1p, Aft2p), two regulators of the oxidative stress response (Yap1p and Skn7p) and certain Yap1p homologues (Yap7p, Cin5p and Cad1p), the regulator of proteasome expression (Rpn4p) and several regulators involved in the general stress response (Hsf1p, Msn2p, Msn4p and Gcn4p). Several transcription factors controlling stress response pathways were themselves found to be upregulated at the mRNA level following selenite treatment (figure 2B). For instance, *YAP1 *and *RPN4 *displayed a remarkable pattern of co-induction. *CIN5 *(*YAP4*), which encodes a Yap1p homologue involved in redox homeostasis and salt tolerance [23], was also induced in our experiments, together with *YRR1*, which encode a zinc-finger protein conferring cell resistance to drugs such as 4-NQO or reveromycine and which is upregulated by oxidizing agents such as MMS or benomyl [4,24-26]. More surprisingly, *PDR1*, which encodes the major regulator of pleiotropic drug resistance in *S. cerevisiae *[13], and *AFT2*, which encodes one of the two main regulators of iron homeostasis [27,28], were both induced by selenite, although no transcriptional regulation of these two genes has ever been reported before. We investigated possible connections between the mechanisms regulating these transcription factors.

### A transcriptional loop connects RPN4 to PDR1

**Figure 3:**
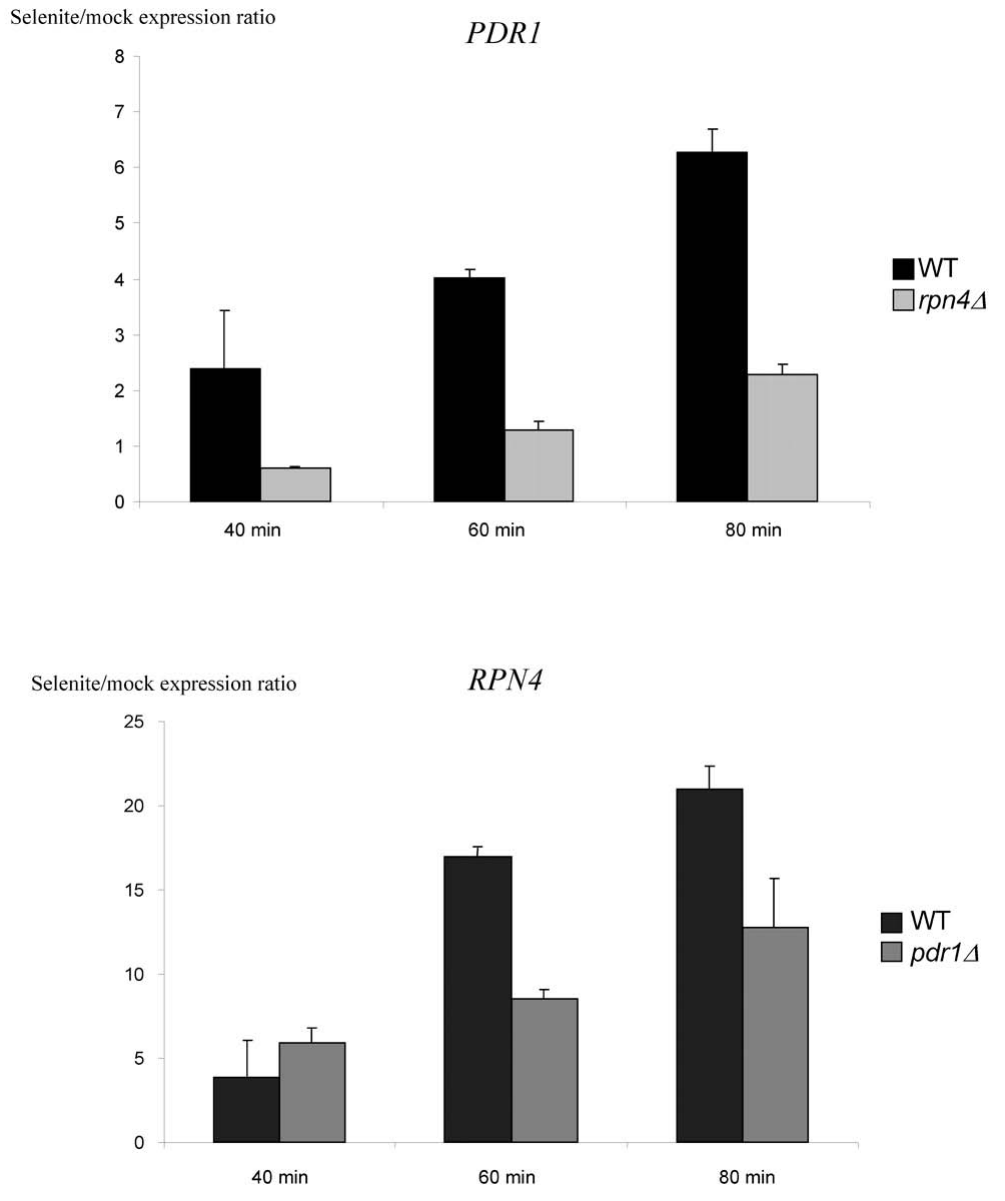
***RPN4 *and *PDR1 *are dependent on each other for regulation by selenite**. The levels of expression of *PDR1 *(upper panel) and *RPN4 *(lower panel) after selenite or mock treatment were quantified in wild-type and *rpn4Δ *(upper panel) or *pdr1Δ *(lower panel) cells, using real-time quantitative PCR. The expression values were normalized using the gene encoding actin (*ACT1*, see methods). The values represented here are the ratios of the normalized expression levels of *PDR1 *(upper panel) or *RPN4 *(lower panel) in the presence of selenite, versus the normalized expression levels of these genes in mock experiments. Each measurement was repeated three times on independent samples. The standard errors are indicated.

We first examined the basis of the regulation of *PDR1 *and *RPN4*. The induction of these two genes by selenite was confirmed by quantitative RT-PCR (figure 3). The induction of *RPN4 *in response to oxidative stress was previously shown to be largely dependent on Yap1p [3,6]. *RPN4 *has also been shown to be activated by gain-of-function alleles of *PDR1 *[12] and the *RPN4 *promoter contains a PDRE which is actually bound by Pdr1p *in vivo *and *in vitro *[2,29]. This PDRE was recently shown to be essential to the full induction of *RPN4 *in response to oxidative stress. This effect was principally attributed to Pdr3p, which also recognizes PDRE [6]. However, *pdr1Δ *cells have normal basal levels of *RPN4 *mRNA and of proteasome activity [29] and *RPN4 *is not induced by drugs which efficiently trigger the Pdr1p/Pdr3p multidrug resistance response [2,4,5]. Finally, the impact of Pdr1p on the *RPN4 *expression in response to stress still had to be established. We therefore examined the expression of *RPN4 *in the presence of selenite in *pdr1Δ *cells (figure 3). We observed a slight decrease of the *RPN4 *induction from 60 minutes after selenite exposure. This result proved that Pdr1p has a modest but significant role in the induction of *RPN4 *in response to oxidative stress.

In searches of SGD [30-32] and Yeastract [33] databases for transcriptional regulators that might account for the selenite-dependent induction of *PDR1*, we identified a conserved Rpn4p recognition element in the promoters of the *PDR1 *orthologues in *Saccharomyces sensu stricto *species. Moreover, Rpn4p was found to bind to the *PDR1 *promoter in a global study of the genomic locations of yeast transcription factors binding sites [30]. We therefore monitored *PDR1 *expression in an *rpn4Δ *strain. The Rpn4p had no apparent role in *PDR1 *basal expression but the inactivation of *RPN4 *severely reduced the sensitivity of *PDR1 *to selenite (figure 3). We conclude from these results that a positive transcriptional loop connects *RPN4 *and *PDR1 *in response to selenite.

### The PDR1/RPN4 loop optimizes the Yap1p dependent oxidative stress response

**Figure 4:**
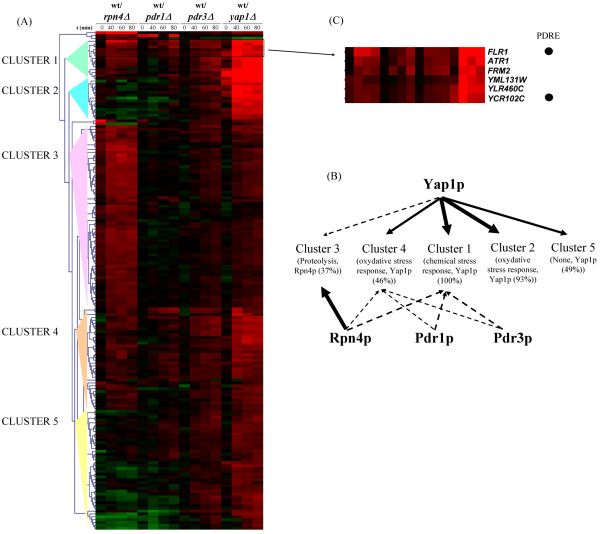
**Respective weights of *RPN4*, *PDR1*, *PDR3 *or *YAP1 *in the selenite response**. (A) Hierarchical clustering of the genes dependent on one or several of the four transcription factors studied for selenite induction. DNA microarrays were used to compare gene expression levels between wild-type and *rpn4Δ*, *pdr1Δ*, *pdr3Δ *or *yap1Δ *exposed to selenite (times 40, 60 and 80 minutes) or mock-treated (time 0). The 175 genes displaying an alteration of selenite induction in at least one mutant strain were clustered into 5 groups (see additional files 5 and 6). (B): Schematic representation of the importance of each transcription factor in the regulation of the five clusters defined in (A). The arrows symbolize the positive regulation of each cluster by the transcription factor: the width of the arrows indicates the importance of the regulation (large arrows: strong effect, thin arrows: weak effect). Dashed arrows indicate that the transcription factor controls the expression of only some of the genes present in the cluster. Solid arrows mean that all the genes present in the cluster are regulated. The relevance of the Gene Ontology categories and regulatory relationships in each of the clusters was investigated with the SGD GO term finder and Yeastract tools [33,34]. Only the main GO category with a p-value < 0.0001 and the main transcription factor known to regulate the genes in one cluster were indicated. (C): Enlargement of the part of cluster 1 containing the genes most sensitive to the deletion of *PDR1 *and *PDR3*. Gene names are indicated. The presence of a PDRE in the promoters of these genes is indicated by black dots.

**Figure 5:**
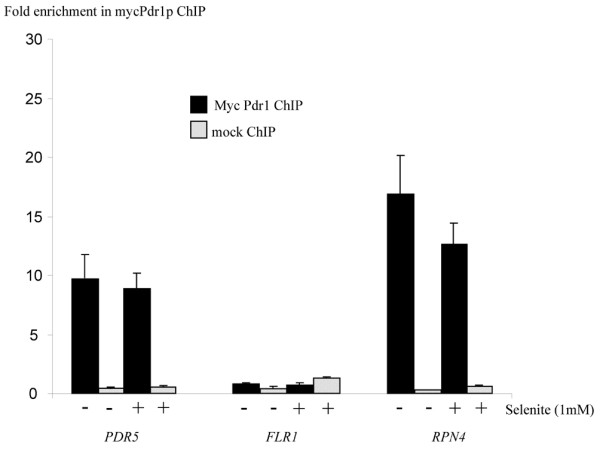
**Pdr1p binds to the promoter of *RPN4 *but not to that of *FLR1 *in the presence of selenite**. The binding of Pdr1p to promoters was assessed by combining chromatin immunoprecipitation with real-time quantitative PCR, using strains harboring a chromosomal tagged version of Pdr1p (myc-Pdr1p). Sequence enrichment in the ChIP (i.e. ChIP/whole cell extract ratio) was normalized using the *ACT1 *ORF as a reference. Similar experiments were conducted on cells with the untagged Pdr1p as a negative control ("mock ChIP"). The *PDR5 *promoter was used as a positive control for Pdr1p binding. The results shown for *FLR1 *were obtained using a pair of oligonucleotides spanning the PDRE motif present in the FLR1 promoter. The cells were exposed to 1 mM of selenite (+) or mock-treated (-) for 60 minutes before the beginning of the ChIP procedure.

We investigated the role of the *PDR1*/*RPN4 *loop, by carrying out a genome-wide analyses of the contributions to the selenite response of Rpn4p, Pdr1p, Yap1p, which controls *RPN4 *expression in response to metalloids [3], and Pdr3p, the functional homologue of Pdr1p. Fluorescent cDNAs from *yap1Δ*, *rpn4Δ*, *pdr1Δ *or *pdr3Δ *cells treated for 0, 40, 60 or 80 minutes with 1 mM of selenite, were hybridized to microarrays together with cDNA from wild type cells subjected to identical treatment (see additional file 5). We then focused on 175 genes displaying levels of induction lower than those observed for the wild type in at least one of the mutants for at least two consecutive time points (see additional file 6). These genes were classified as a function of their gene expression patterns using hierarchical clustering (see additional file 6). Based on the clustering tree obtained, we defined five groups corresponding to five different patterns of expression (figure 4A). We used Yeastract [33] and the SGD GO term finder [34] to address the main transcriptional regulators and functional annotations available for these genes (figure 4B). Yap1p was the factor regulating the largest number of these genes (about 80%), which were, as expected, mostly involved in redox homeostasis and the chemical stress response (clusters 1, 2, 4 and 5). Many of these genes had already been annotated as Yap1p targets and their selenite induction was abolished in the absence of *YAP1 *(clusters 1 and 2). Yap1p also had a slight effect on some genes involved in proteolysis (part of cluster 3), possibly indirectly due to the significant influence of Yap1p on *RPN4 *expression (see additional file 6). *RPN4 *deletion had an effect on about 60% of the genes shown in figure 4A. Most of these genes encoded proteins involved in proteolysis (cluster 3), as expected, and one third of them had already been identified as Rpn4p targets (figure 4B). More surprisingly, the deletion of *RPN4 *slightly but reproducibly decreased the selenite induction of several Yap1p targets (clusters 1 and 4). The inactivation of *PDR1 *or *PDR3 *altered the expression of few genes (less than 20% of the genes in figure 4A), all of which were Yap1p targets (upper parts of clusters 1 and 4) and encoded proteins involved in chemical stress response and xenobiotic export (*FLR1*, *ATR1*, *FRM2*, etc.). Remarkably, these effects were similar to those of the *RPN4 *deletion on these genes (figure 4C). We carried out chromatin immunoprecipitation (ChIP) analyses, together with real-time quantitative PCR, to investigate the direct binding of Pdr1p to these Pdr1p-dependent genes, using a myc-tagged version of Pdr1p [2]. We used the *PDR5 *promoter as a positive control, as this sequence constitutively binds Pdr1p [2]. We first focused on the *FLR1 *promoter, which contains a PDRE and is partially controlled by Pdr3p in response to oxidative stress [35]. We detected no significant binding of Pdr1p to the promoter of *FLR1*, despite using several pairs of oligonucleotides to scan the entire *FLR1 *promoter region (figure 5). Similarly, the promoters of *ATR1*, *FRM2 *and *YCR102c *did not bind by Pdr1p in the presence of selenite (data not shown). *RPN4 *was the only gene tested that displayed Pdr1p-dependent selenite induction, its promoter binding directly to Pdr1p (Figure 5).

### Conservation of the YAP1/RPN4/PDR1 co-regulation in C. glabrata

**Figure 6:**
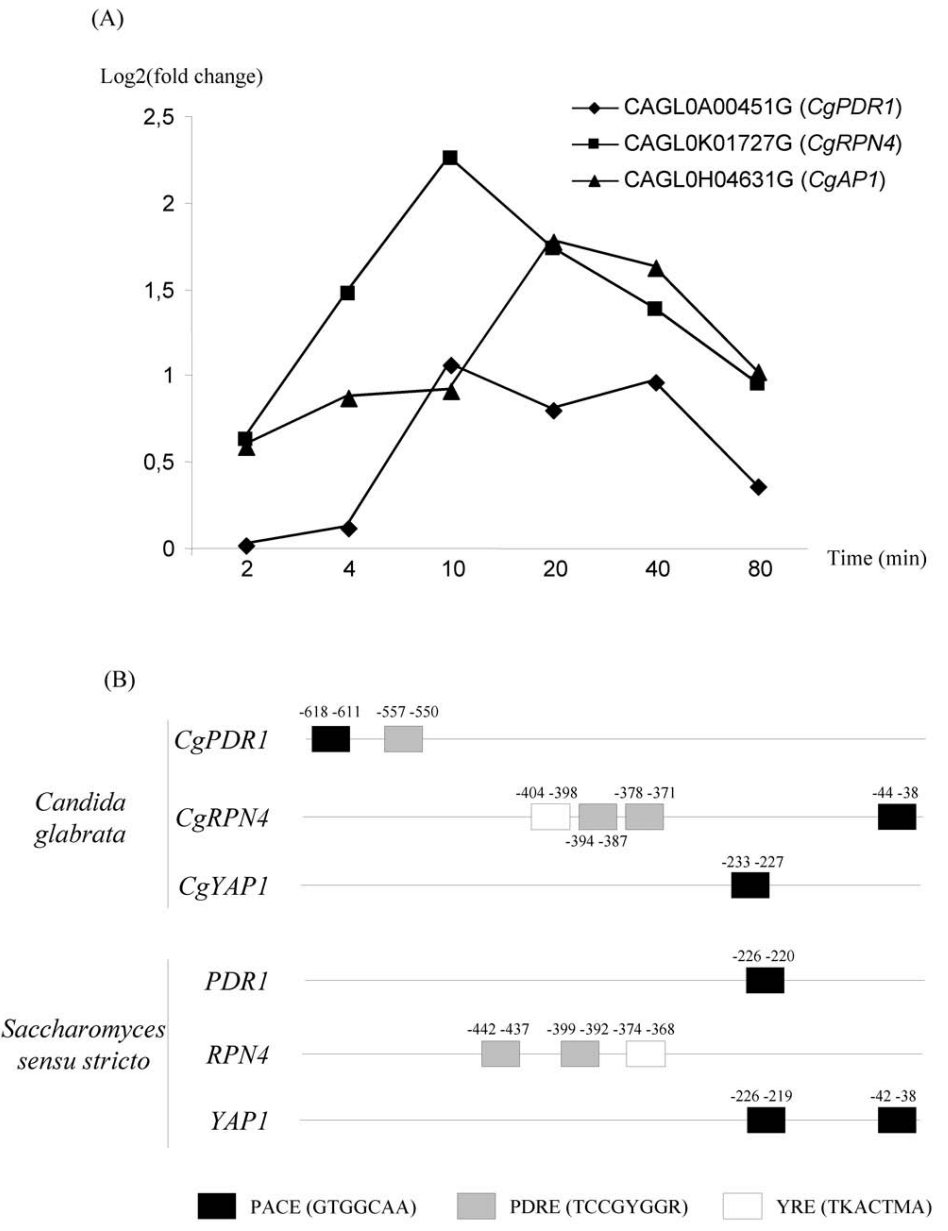
***PDR1*, *RPN4 *and *YAP1 *are induced by oxidative stress in *C. glabrata***. (A) DNA microarrays were used to analyze the transcriptome response of *C. glabrata *cells to the oxidizing agent benomyl. The data obtained were analyzed and discussed fully in another manuscript (Lelandais et al., in preparation). (B) We searched for consensus DNA binding sites for Rpn4p, Yap1p and Pdr1p in the *CgRPN4, CgAP1 *and *CgPDR1 *promoters using RSA tools software [65]. The positions of the motifs identified are indicated relative to the start codon of the corresponding ORF. The *Saccharomyces sensu stricto *species used here are *S. cerevisiae*, *S. paradoxus*, *S. bayanus*, *S. kuudriazvezii *and *S. mikatae*. The positions of the motifs indicated for these species refer to the *S. cerevisiae *genes.

We assessed the conservation of *RPN4*, *PDR1 *and *YAP1 *co-regulation in the pathogenic yeast *C. glabrata*. This species does not belong to the *Saccharomyces sensu stricto *group but is much more closely related to *S. cerevisiae *than the other pathogenic *Candida *species [36]. Especially, clear *C. glabrata *orthologs of *PDR1 *(called *CgPDR1*), *YAP1 *(*CgAP1*) and *RPN4 *(*CgRPN4*) could be defined, whereas there seems to be no ortholog of *PDR3 *in this species [37]. As in *S. cerevisiae*, *CgRPN4 *is upregulated in strains harboring gain-of-function alleles of *CgPDR1 *[38]. We used *C. glabrata *microarrays to investigate the expression patterns of *CgAP1*, *CgRPN4 *and *CgPDR1 *in response to the oxidative stress caused by the antifungal drug, benomyl (figure 6A). A complete comparison of the oxidative stress responses of *C. glabrata *and *S. cerevisiae *is presented elsewhere (Lelandais et al., manuscript in preparation). We observed that benomyl induced the *C. glabrata *homologues of *YAP1*, *RPN4 *and *PDR1 *(figure 6A). We therefore analyzed the DNA regions upstream from the *CgRPN4, CgAP1 *and *CgPDR1 *ORFs (figure 6B). As in *S. cerevisiae*, two PDRE and one YRE were found in the *CgRPN4 *promoter, whereas one PACE was found in the *CgPDR1 *and *CgAP1 *promoters (figure 6B). These data strongly suggest that the cross-regulation of *PDR1 *and *RPN4 *on one hand, and of *YAP1 *and *RPN4 *on the other, is conserved from *S. cerevisiae *to *C. glabrata*. These findings are consistent with this network playing a role in the fine-tuning of oxidative stress responses in yeasts.

### Expanding the Yap1 network: connections with the Yrr1 and Aft2 regulons

**Figure 7:**
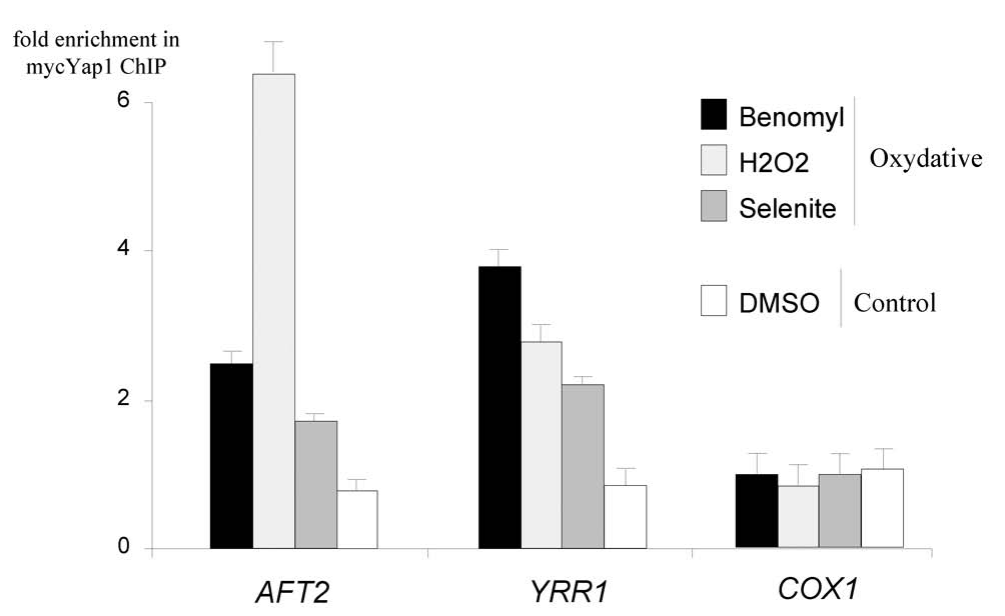
**Yap1p binds to the promoters of *YRR1 *and *AFT2 *in oxidative conditions**. The global DNA binding pattern of Yap1p was determined by chromatin immunoprecipitation using strains harboring a tagged version of Yap1p (myc-Yap1p). The immunoprecipitate DNA was hybridized with intergenic DNA microarrays, together with genomic DNA (see methods). The cells were previously treated with benomyl, hydrogen peroxide or selenite (oxidative conditions); or with DMSO (control condition). Enrichment ratios were normalized using the print-tip median method (see methods). Each experiment was carried out four (oxidative conditions) or two (control condition) times. The histograms indicate the enrichment of the sequence in the immunoprecipitate, normalized with respect to the value obtained for whole cell extract. The results obtained for the mitochondrial ORF *COX1 *are shown as a negative control. The standard errors are indicated. The full results are shown in additional file 11.

The *YRR1 *and *AFT2 *genes were both induced by selenite (figure 2). *YRR1 *was shown to be regulated by oxidizing agents such as benomyl or mancozeb in a Yap1p-dependent manner [4,39]. No transcriptional regulation of *AFT2 *has ever before been reported. The selenite-driven expression of *AFT2 *was weaker in the *yap1Δ *strain than in the wild type (see additional file 6 and cluster 5 of figure 4A) and one YRE (TTAGTCA) conserved in *Saccharomyces sensu stricto *species, was found in the promoter of this gene 147 base pairs upstream from the ATG codon [31,32]. We conducted genome-wide location analyses of a myc-tagged version of Yap1p [40] using the ChIP-chip technique [30]. Previous experiments indicated that Yap1p could discriminate between different sources of oxidative stress but that its DNA-binding properties were independent of the oxidizing agents used [4,41]. We therefore increased the relevance of our ChIP-chip data, by carrying out analyses of three different sources of oxidative stress: selenite (1 mM), hydrogen peroxide (0.3 mM) and benomyl (20 μg/ml). The doses of hydrogen peroxide and benomyl and the time of exposure (20 minutes) used were chosen based on previous experiments [4,7,40]. Each experiment was carried out independently four times. We used the SAM method [21] to obtain lists of significantly and reproducibly bound targets in the three sets of conditions. We considered as potential targets only those sequences bound in all the oxidative conditions tested. We controlled for false positive by conducting similar experiments in control conditions, on cells treated with DMSO. In these conditions, Yap1p was efficiently excluded from the nucleus [42]. We then deleted the DNA sequences which were significantly enriched in the DMSO experiments, to obtain the final list of Yap1p direct targets presented in the additional file 7. We identified 310 DNA sequences, corresponding to about 300 promoter regions, that were reproducibly and significantly enriched in all conditions. According to the SGD GO term finder [34], the genes regulated by these promoter regions were mostly involved in the cell response to chemical stimuli (p = 2.21 × 10^-18^), oxidative stress (p = 1.16 × 10^-9^), and drugs (p = 1.35 × 10^-3^); and in sulfur metabolism (p = 4.1 × 10^-4^). This list contained many genes previously known to be regulated by Yap1p (e.g. *TRX2*, *FRM2*, *TRR1*, *TSA2*, *SOD1*, etc.), including *CIN5 *and *RPN4 *(see additional file 7). Remarkably, 75% of these 310 sequences contained a YRE (see additional file 7) but only 23% of them were induced by selenite in a Yap1p-dependent manner, which confirmed that Yap1p binding is necessary but not sufficient for transcriptional regulation [4,30,41]. Our ChIP-chip results overlapped with the clusters defined in figure 4. The groups of genes which were highly dependent on Yap1p for selenite induction (clusters 1 and 2), contained 72% and 86%, respectively, of sequences binding Yap1p in our experiments. Clusters 4 and 5 included only 50% and 33% of sequences binding Yap1p. Only 5% of the genes in cluster 3, which was mainly composed of targets of Rpn4p, were found to bind Yap1p, confirming that the mild effect of Yap1p on the expression of about half of this group is indirect. Finally, the sequences corresponding to the *YRR1 *and *AFT2 *promoters were significantly and specifically enriched in all the oxidative conditions tested (Figure 7). Based on these data, we conclude that the selenite-dependent induction of *YRR1 *and *AFT2 *is directly controlled by Yap1p.

## Discussion

### Network mapping for the selenite response in budding yeast

We used selenite as a model stress to decipher part of the transcriptional network controlling the adaptation of the genome expression to toxic environmental conditions in yeast. Our data suggested that one of the earliest effects of selenite on gene expression was the induction of an iron starvation-like response, known to be controlled by the Aft1p and Aft2p transcription factors [28,43]. Selenite may affect iron homeostasis at two, non exclusive, levels. First, selenium can interact with iron, with a high affinity, in the culture medium [44,45], rendering this metal unavailable to the cell. Second, it may interfere with iron homeostasis by replacing sulfur in iron-sulfur cluster protein biosynthesis in the mitochondria [46]. Interestingly, the sets of yeast mutant strains which accumulating selenium or iron are very similar, suggesting that these two elements are metabolized through similar cellular routes and affect similar cellular processes [47]. Like arsenite [3], selenite induces a strong oxidative stress response, triggering redox homeostasis pathways and proteasome activity. Selenite may affect redox homeostasis in several ways. First, each selenite molecule contains three atoms of oxygen, which may generate reactive oxygen species (ROS) during selenite reduction. Second, selenite is metabolized through interaction with thiol derivatives, including gluthatione [19], probably leading to imbalance in redox homeostasis. Third, as mentioned above, selenite may interfere with iron homeostasis and iron-sulfur cluster protein biosynthesis, potentially affecting mitochondrial activity and redox homeostasis [48]. Another feature of the oxidative stress response generated by metals and metalloids is the upregulation of genes involved in methionine and sulfur metabolism [3,49]. This effect was not detected in our transcriptome analyses of the yeast response to selenite. This was certainly due to our experiments being carried out in rich media, in which the abundance of sulfur amino acids efficiently switched off the MET gene transcription [50], whereas the studies mentioned above were conducted in minimal media. Despite the simplicity of its molecular structure, selenite induces many different stress response pathways. Moreover, the dose of selenite used clearly compromised the cells ability to respond to stress efficiently. We were therefore able to observe the regulation of mRNA levels for many transcriptional regulators involved in stress responses. This made it possible to reveal new connections between these stress response pathways.

### Transcriptional loops connect proteasome to pleiotropic drug resistance and oxidative stress response

The proteasome functions in many cellular processes, some of which are essential for cell survival as cell cycle progression or the adaptation to environmental changes [9,51]. Its activity is therefore tightly regulated. One level of regulation is the expression of its subunits, which is controlled by the Rpn4p transcription factor in yeast [52]. *RPN4 *is itself positively regulated by a complex array of transcriptional controls connected to environmental stress. These include the heat shock factor Hsf1p, the multidrug resistance regulators Pdr1p and Pdr3p and the oxidative stress response major regulator, Yap1p [3,6]. In this work, we confirmed that Pdr1p and Yap1p were required for the full selenite-driven induction of *RPN4 *(figure 3 and see additional file 6). Recent data have suggested that *RPN4 *not only is a target for stress response pathways but Rpn4p also has a direct impact on the expression of some of its regulators. Indeed, the response of *YAP1 *to selenite and arsenite was diminished in the absence of *RPN4 *(see additional file 6,[3]), as Rpn4p binds to a PACE present in the *YAP1 *promoter [30], providing strong evidence in favor of a transcriptional loop connecting *RPN4 *and *YAP1*. Similarly, one of the major findings of this study was that Rpn4p controls the expression of *PDR1 *in response to selenite (figure 3). Therefore, *RPN4 *establishes positive feedback loops with both the oxidative stress response and the pleiotropic drug resistance network. These loops seem to optimize part of the oxidative stress response, as the deletion of *RPN4*, *PDR1 *or *PDR3 *decreases the positive effect of Yap1p on some of its target genes by 50% (figure 4C). These effects on gene expression have apparently no impact on cell survival in laboratory conditions (see additional file 8), but evidence for their biological significance is provided by the observation of *RPN4*, *YAP1 *and *PDR1 *co-regulation in response to oxidative stress in the yeast *C. glabrata *(figure 6A). Our results are also consistent with those of a recent study showing that the Rpn4p, Yap1p, Pdr3p and Yrr1p transcription factors collaborate in the upregulation of *FLR1 *in response to oxidative stress [39]. Positive feedback loops may induce bistability in biological systems [53]. Bistability has obvious advantages in the responses of microbial cells to environmental changes. After transient exposure to stress, bistability allows some cells to maintain a particular pattern of gene expression long after the stimulus has ended. This provides a mechanism for the anticipation of future environmental changes based on past environmental conditions and can potentially generate heterogeneity in isogenic cell populations by generating bimodal population responses [53,54]. For Rpn4p and Yap1p, the transcriptional positive feedback loop is counterbalanced by a post-translational negative feedback loop, with the levels of Rpn4p and Yap1p being negatively regulated by the proteasome [11,55].

### A third mode of functioning for the PDR pathway

**Figure 8:**
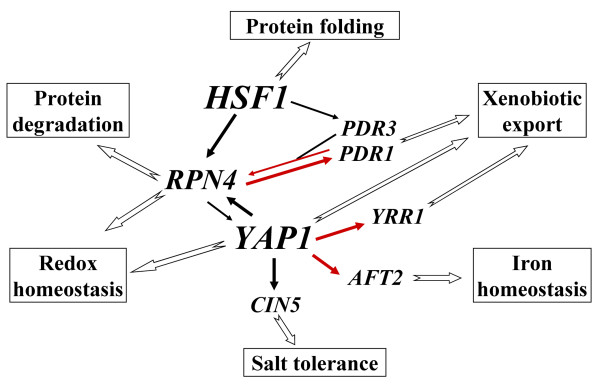
**A highly interconnected transcriptional network is involved in the response to selenite**. This diagram indicates the regulatory interactions involved in the response to selenite, as identified through this study. All the arrows represent positive transcriptional regulations of transcription factor encoding genes or of groups of target genes (symbolized then by functional categories) by a transcription factor. Red arrows indicate interactions demonstrated for the first time by this work. The arrows in bold indicate high-weighted interactions and the thin arrows symbolize low-weighted interactions, according to the results of this study (in the case of Rpn4p, Yap1p, Pdr1p, Pdr3p) or measurements published by Hahn et al (in the case of Hsf1p) [6].

Two modes of functioning of the PDR pathway have been extensively described. First, Pdr1p and Pdr3p are both involved in the basal expression and drug-dependent up-regulation of multidrug resistance transporter genes, such as *PDR5*, *SNQ2 *and *YOR1 *[13]. Second, Pdr3p is involved in a retrograde response negatively connecting the expression of drug resistance transporters to the mitochondrial respiratory activity [56,57]. The tight connection described above between *RPN4 *and *PDR1 *reflects a third mode of functioning for the PDR pathway. In response to oxidative stress, Pdr1p and Pdr3p control the induction of *RPN4 *(figure 3, [6]), which in turn affects the expression of Yap1p target genes such as *FLR1 *(figure 4, [35]). Remarkably, *FLR1 *and *RPN4 *were not induced by drugs that efficiently trigger the "classic" PDR response, such as progesterone or fluphenazine [2,5], or by mitochondrial defects that stimulate Pdr3p activity [57]. *PDR1 *and *PDR3 *also had original patterns of regulation in response to selenite. As described above, *PDR1 *is under the control of Rpn4p (figure 3). By contrast, *PDR3 *has been reported to be controlled by Hsf1p in response to oxidative stress [6]. The induction of *PDR3 *by selenite (see additional file 9) together with the other targets of Hsf1p (see additional file 4), suggests that the regulation of *PDR3 *by Hsf1p is effective in the presence of selenite (figure 8).

### Yap1p: a central node in the oxidative stress network

Yap1p is the major regulator of the genes encoding proteins involved in redox homeostasis in response to various sources of oxidative stress [1,58]. Yap1p has been shown to influence other regulatory pathways, for instance by regulating *RPN4 *and *CIN5 *[3,23]. A previous global analysis of DNA binding sites for most of the transcription factors of yeast provided the first evidence to suggest that Yap1p may regulate the expression of transcription factors [30]. We have shown that the induction of *YRR1 *[4] and *AFT2 *(figure 4) in response to stress is dependent on *YAP1 *and that Yap1p actually binds to the promoters of these genes in the presence of various sources of oxidative stress (figure 7). In response to both benomyl and selenite, *YRR1 *was induced later than other Yap1p targets (figure 2, [4]). Remarkably, several Yrr1p targets (e.g. *FLR1*, *SNG1*, *SNQ2*) [25] were found to be also directly regulated by Yap1p in response to selenite (see additional files 6 and 7). The role of Yrr1p therefore seems to be to support Yap1p in the long-term regulation of these genes. The physiological connection between iron metabolism and redox homeostasis has been established before. In particular, it has been shown that the activity of the Aft1p transcription factor, which senses iron through iron-sulfur protein biogenesis status [46], is influenced by the glutathione biosynthesis pathway and the Grx3 and Grx4 glutaredoxins, which are involved in the thiol redox system [48]. Moreover, the double deletion of *AFT1 *and *AFT2 *induces cell hypersensitivity to oxidative stress [27]. Our finding that Yap1p controls *AFT2 *expression provides a direct transcriptional connection between the two pathways. It may seem paradoxical that Yap1p positively controls a system that responds to iron starvation, given that iron uptake is likely to cause oxidative stress. Noteworthy, Aft2p specifically controls the expression of genes involved in the transport of iron from the cytosol to the vacuole and mitochondria [28]. By contrast, Aft1p, which actually controls iron uptake from the environment, does not seem to be positively regulated by Yap1p.

In conclusion, Yap1p is a central node in the oxidative stress response network, coordinating the expression of at least four transcription factors involved in various stress response pathways (figure 8).

#### Structure and dynamics of the regulatory networks driving cell adaptation to environmental changes

We analyzed the structure and dynamics of the transcriptional regulatory network which controls the adaptation of yeast transcriptome to toxic doses of selenite (figure 8). Our findings hinted several important features of the regulatory networks involved in chemical stress responses. First, these networks are highly interconnected. The cross-regulation of different regulators makes it possible to transmit information between the different transcriptional routes, resulting in the tight coordination of the various cellular pathways required for cell survival. Second, these networks have versatile and dynamic structures and properties. This plasticity is based on the combination of different transcription factors responsive to different chemical and physical parameters, but also on the fact that the same transcription factor can change its protein partners and/or its DNA binding properties to adapt its activity to the physiological conditions (e.g. the three modes of functioning of the Pdr1p/Pdr3p combination). Third, there is a clear hierarchy in these networks, as illustrated by unidirectional regulations (e.g. Hsf1p on *RPN4*) and the unequal weightings of different relationships (e.g. *RPN4*/*YAP1 *and *RPN4*/*PDR1 *loops), which may also be a function of time and physiological status of the cell.

## Methods

### Yeast strains

The *Saccharomyces cerevisiae *strains used were all of the BY4742 (*MATa*; *his3Δ1*;*leu2Δ0; lysΔ0; ura3Δ0*) background. The *rpn4Δ*, *yap1Δ*, *pdr1Δ *and *pdr3Δ *strains were purchased from Euroscarf [59]. The Pdr1-myc and the Yap1-myc strains have been described elsewhere [2,40]. The *Candida glabrata *strain was CBS418.

### Growth conditions and time-course analyses of stress responses

Cells were grown at 30°C in YPD (1%(w/v) bacto-yeast extract, 2% (w/v) bacto-peptone, 2% (v/w) glucose) to an OD_600 nm _of 0.5. The cultures were then split in two. Sodium selenite (1 mM), hydrogen peroxide (0.3 mM) or benomyl (20 μg/ml) was added to one of the two half-cultures and water or DMSO (mock treatment) was added to the other. The cells were incubated for an appropriate period of time (see text), and were then either flash-frozen in cold ethanol for RNA extraction or treated with formaldehyde for chromatin immunoprecipitation (see below).

### RNA extractions

Cell culture (15 ml) was flash-frozen in 30 ml of absolute ethanol at -80°C. The cells were harvested by centrifugation (4 minutes at 3000 g). The cell pellets were stored at -80°C. Total RNAs was extracted as previously described [2].

### Transcriptome and quantitative RT PCR analyses

The *S. cerevisiae *microarrays used are fully described in Array express ([60]; accession numbers A-MEXP-337, A-MEXP-114 and A-MEXP-1064). The *C. glabrata *microarrays are described in the Gene Expression Omnibus database ([61]; accession number: GPL3922). We used 10 μg of total RNA for cDNA synthesis and labeling. The microarray experiments were conducted as previously described [2]. Raw data were normalized using global lowess followed by print-tip median methods, with background removal, as implemented in Goulphar [62]. Experiments with wild-type strains were carried out 4 times, with dye swapping. The statistical significance of the differences in expression observed was determined with the TMEV version of SAM, with a FDR of 5% and the exact number of permutations [21,63,64]. Only genes with less than 25% missing values were considered for the SAM analyses. The remaining missing values were imputed by the KNN input method directly in the TMEV application [21,63,64]. Hierarchical clustering was performed using TMEV, with Euclidean distances and average linkage [63,64]. The complete *S. cerevisiae *transcriptome data are available as additional files 1 and 5. The raw data can be downloaded from the array express database (accession number: E-TABM-439). The *C. glabrata *data can be downloaded from the Gene Expression Omnibus database (accession number: GSE10244).

We used 500 ng of total RNA for quantitative RT-PCR, which was performed as previously described [2]. The oligonucleotides used are described in additional file 10. *ACT1 *was used as a reference for normalization.

### Chromatin immunoprecipitation

Chromatin immunoprecipitation experiments, followed by intergenic microarray or quantitative PCR analyses, were performed as previously described [2]. The *S. cerevisiae *intergenic arrays are described in the Array express database (accession number: A-MEXP-1065). The array results were normalized using the print-tip median [62]. The statistical significance of the ChIP enrichments was assessed with the TMEV version of SAM with a FDR of 1% and the exact number of permutations [21,63,64]. The complete ChIP-chip results are available as additional file 11. The raw data can be downloaded from the array express database (accession number: E-TABM-437). The sequence of the oligonucleotides used for quantitative PCR can be found in additional file 10.

### Data mining

Functional analyses and network mapping of the genome-wide data were carried out with T-profiler [22], SGD GO term finder [32] or Yeastract [33], using the default parameters. Promoter sequence analyses were performed with the DNA pattern search tool from RSA tools [65] and the genome Browser tool from the SGD [66].

## Abbreviations

ESR: environmental stress response; YRE: Yap1p response element; PACE: proteasome associated control element; PDRE: pleiotropic drug response element; ORF: open reading frame; GO: gene ontology; MMS: methyl methane sulfonate; 4-NQO: nitroquinoline oxide; DMSO: dimethyl sulfoxyde; SGD: saccharomyces genome database; ChIP: chromatine immunoprecipitation; ChIP-chip: ChIP associated to microarrays; SAM: significance analysis of microarrays; ROS: reactive oxygen species; MET: methionine biosynthesis pathway; PDR: pleiotropic drug resistance.

## Authors' contributions

HS, VF and VT carried out the transcriptome and Yap1p DNA binding experiments. EP performed the ChIP analyses of Pdr1p DNA binding. GL and SL contributed to the bioinformatic analyses of transcriptome and ChIP-chip results. CJ contributed to the writing of the manuscript. FD directed the work, carried out the transcriptome analyses of mutant strains, analyzed the data and wrote the manuscript.

## Supplementary Material

Additional file 1 **Transcriptome analyses of the response to selenite in wild-type *S. cerevisiae *cells**. The ORF names are indicated, according to the SGD annotation. For each ORF, the numbers are the median Log2 values of the ratio of expression between treated and untreated cells at various time points, calculated from four independent experiments. The SAM results are indicated as follows: NS: non significant; NA: not applicable (less than 3 independent measurements available); S: significant. At 2 and 5 minutes, no variation in gene expression was considered significant by SAM. We therefore indicate no SAM results for these time points. Click here for file

Additional file 2 Number of genes significantly up-or downregulated for two consecutive time points in the selenite response. Click here for file

Additional file 3 **T-profiler results**. Upper part: The gene ontology categories displaying significant changes in expression in response to selenite were identified by t-profiler analysis [22]. The t-values are indicated. Only T-values in bold were significant (E-value < 0.05). Only GO categories significant for at least two consecutive time points are presented in this table. Lower part: The transcription factors with ChIP-chip target genes [30] displaying a significant change in expression during the selenite response were identified by t-profiler [22]. The t-values are indicated. Only T-values in bold were significant (E-value < 0.05). Only transcription factors significant for at least two consecutive time points are presented in this table. Click here for file

Additional file 4 **Gene expression patterns for Gene Ontology categories identified by t-profiler, following exposure to selenite**. Wild-type cells were treated with selenite and gene expression levels were evaluated by microarray analysis, using untreated cells as a reference. Note that the lists of genes given is not exhaustive and corresponds to a sample of the genes present in these GO categories. More complete information can be found in additional file 1. Click here for file

Additional file 5 **Transcriptome analyses of the response to selenite in mutant cells**. The ORF names are indicated, according to the SGD annotation. For each ORF, the numbers are the median Log2 values of the ratio of expression between wild-type and mutant cells at different time points, calculated from two independent experiments. NA: not available. Click here for file

Additional file 6 **Expression values associated with the eisengram in Figure **4A. The gene and PRF names are indicated, according to the SGD annotation. Genes are ordered according to the hierarchical cluster of figure 4A. For each gene, the numbers are the median Log2 values of the ratio of expression between wild-type and mutant cells at different time points, calculated from two independent experiments. Click here for file

Additional file 7 **Myc-Yap1p ChIP-chip results**. The global DNA binding pattern of Yap1p was determined by chromatin immunoprecipitation using strains harboring a tagged version of Yap1p (myc-Yap1p). The immunoprecipitate obtained was hybridized with intergenic DNA microarrays, together with whole cell genomic DNA (see methods). The cells were previously treated with benomyl, hydrogen peroxide or selenite (oxidative conditions), or with DMSO (reducing condition). SAM was used to sequences significantly enriched in each set of conditions. This table lists the features significantly enriched in all oxidative conditions but not in the reducing condition (negative control). The genes which are potentially under the transcriptional control of the corresponding DNA regions are indicated, based on a previous annotation [30]. The presence of YRE in the corresponding sequences and the presence of the regulated genes in one of the five clusters defined in figure 4 are indicated. Click here for file

Additional file 8 **Phenotype of strains deleted for *RPN4*, *PDR1*, *PDR3 *or *YAP1 *in presence of selenite**. The cells were grown in YPD to an OD_600 nm _of 0.25 (early exponential phase). They were then treated by 0, 0.5, 0.75 or 1 mM of sodium selenite. The graphs represent the OD_600 nm _(Y axis) as a function of the time of exposure to selenite (X-axis). The wild type, *pdr1Δ *and *pdr3Δ *strains exhibited the same sensitivity to selenite in these conditions. The *rpn4Δ *and *yap1Δ *strains were more sensitive than the wild type and this defect is more severe in the case of *yap1D *cells. Click here for file

Additional file 9 **Expression profile for *PDR3 *in response to selenite in wild-type yeast cells**. Levels of *PDR3 *expression were quantified in wild-type cells, by real-time quantitative PCR. Expression values were normalized, using the gene encoding actin (*ACT1*, see methods). The values shown here are the ratios of normalized levels of *PDR3 *expression in the presence of selenite to normalized levels of expression of this gene in mock experiments. Each measurement was repeated three times, on independent samples. The standard errors are indicated. Click here for file

Additional file 10 Oligonucleotides used for quantitative PCR analyses of gene expression or transcription factor DNA binding. Click here for file

Additional file 11 **Genome-wide chromatin immunoprecipitation of Yap1p in different conditions**. The ORF names are indicated, according to the SGD annotation. The intergenic regions are annotated as follows: iORFX is the intergenic region 5' to ORF X, when ORF X is on the Crick strand and 3' when ORF X is on the Watson strand. For each feature, the numbers are the median Log2 values of the ratio of expression between the immunoprecipitate and the whole cell extract, calculated from four independent experiments. Standard deviations (SD) are indicated. NA: value not available. Click here for file
