# Supplementary figures and images for: Structure and properties of transcriptional networks driving selenite stress response in yeasts
Source: BMC Genomics. 2008 Jul 15;9:333. doi: 10.1186/1471-2164-9-333 (PMC2515152; doi:10.1186/1471-2164-9-333)

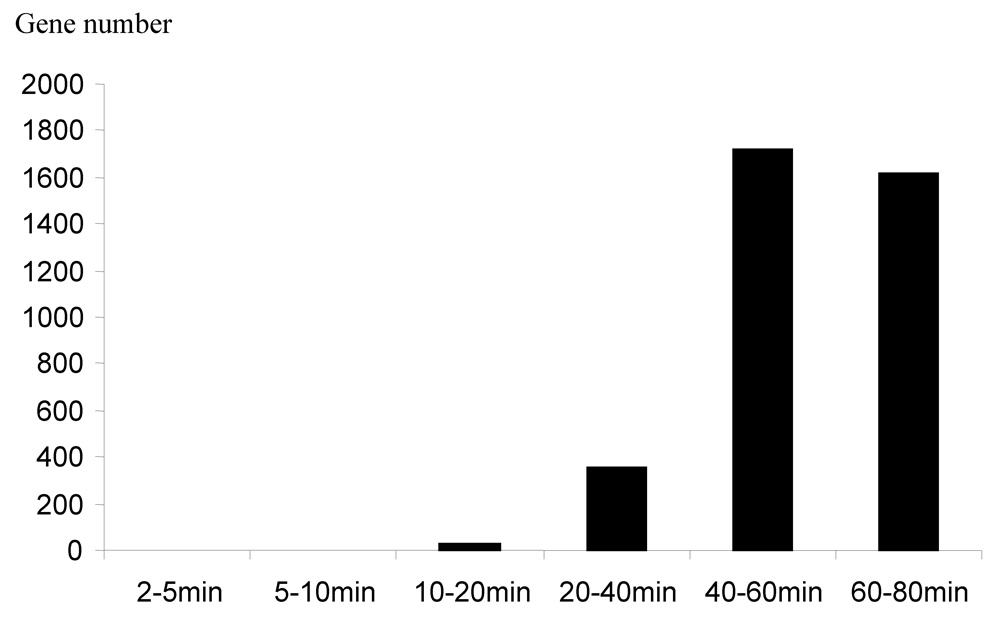

Supplement: Additional file 2 — Number of genes significantly up-or downregulated for two consecutive time points in the selenite response. [file 1471-2164-9-333-S2.tiff]

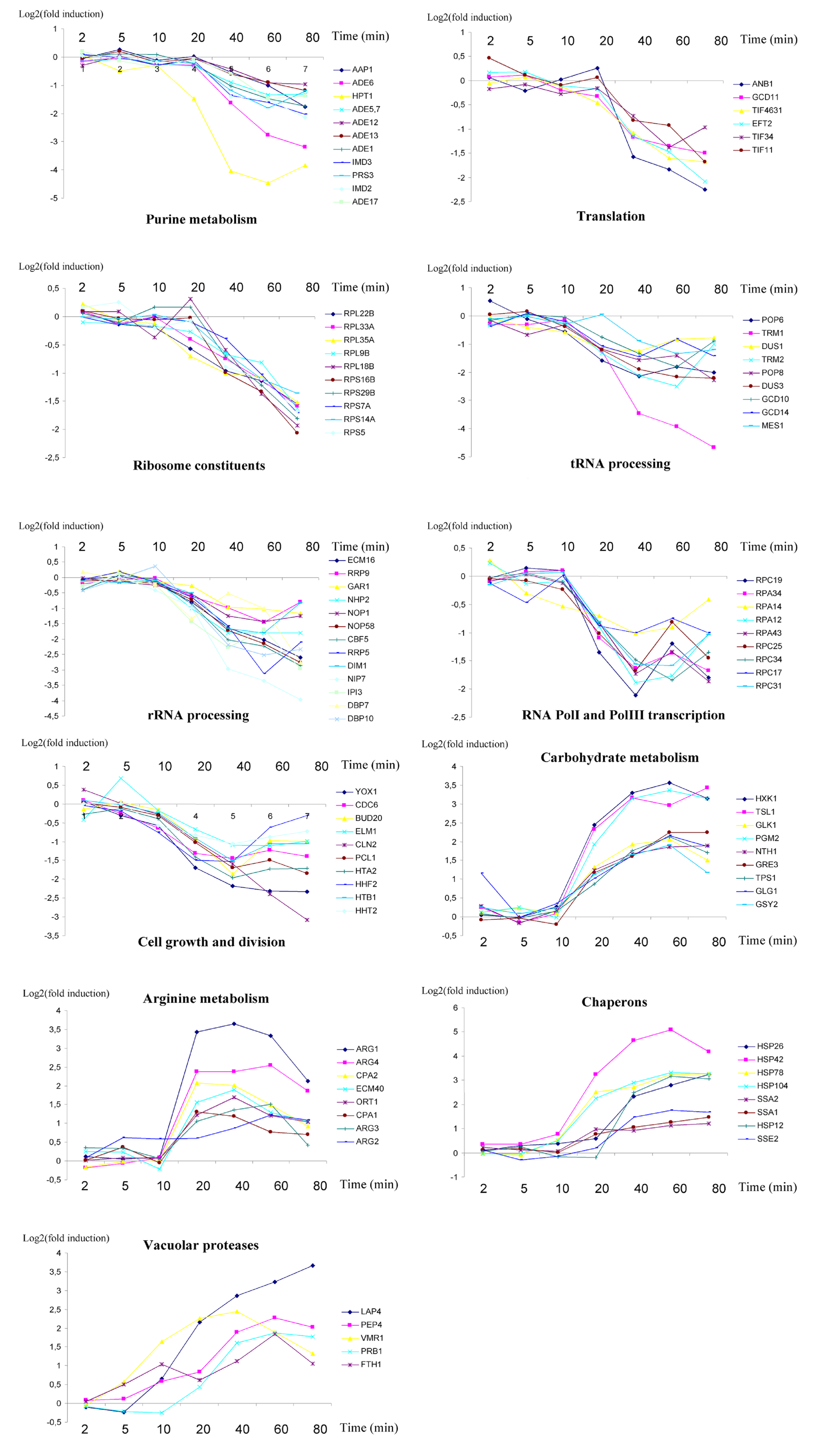

Supplement: Additional file 4 — Gene expression patterns for Gene Ontology categories identified by t-profiler, following exposure to selenite. Wild-type cells were treated with selenite and gene expression levels were evaluated by microarray analysis, using untreated cells as a reference. Note that the lists of genes given is not exhaustive and corresponds to a sample of the genes present in these GO categories. More complete information can be found in additional file 1. [file 1471-2164-9-333-S4.tiff]

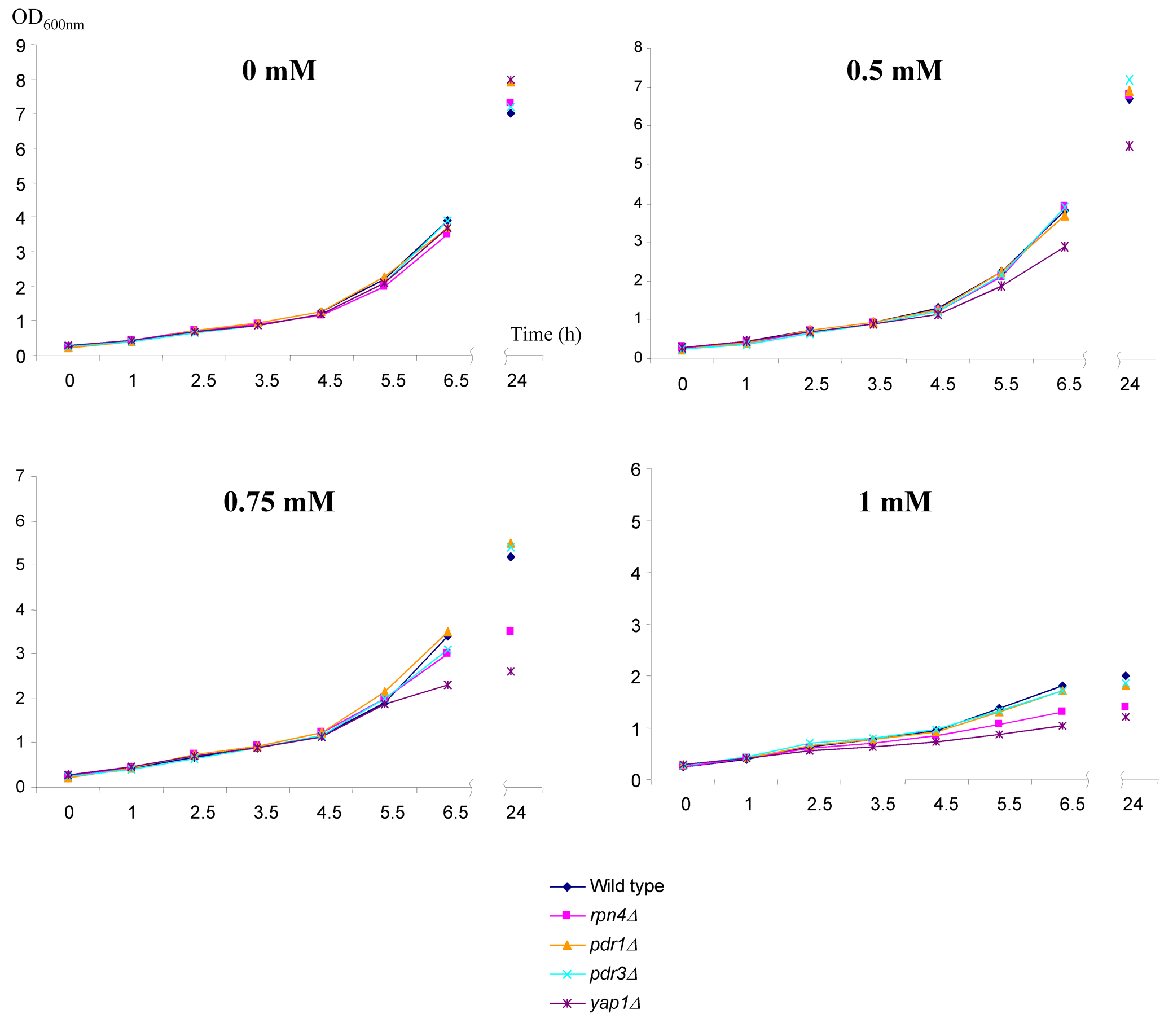

Supplement: Additional file 8 — Phenotype of strains deleted for RPN4, PDR1, PDR3 or YAP1 in presence of selenite. The cells were grown in YPD to an OD600 nm of 0.25 (early exponential phase). They were then treated by 0, 0.5, 0.75 or 1 mM of sodium selenite. The graphs represent the OD600 nm (Y axis) as a function of the time of exposure to selenite (X-axis). The wild type, pdr1Δ and pdr3Δ strains exhibited the same sensitivity to selenite in these conditions. The rpn4Δ and yap1Δ strains were more sensitive than the wild type and this defect is more severe in the case of yap1D cells. [file 1471-2164-9-333-S8.tiff]

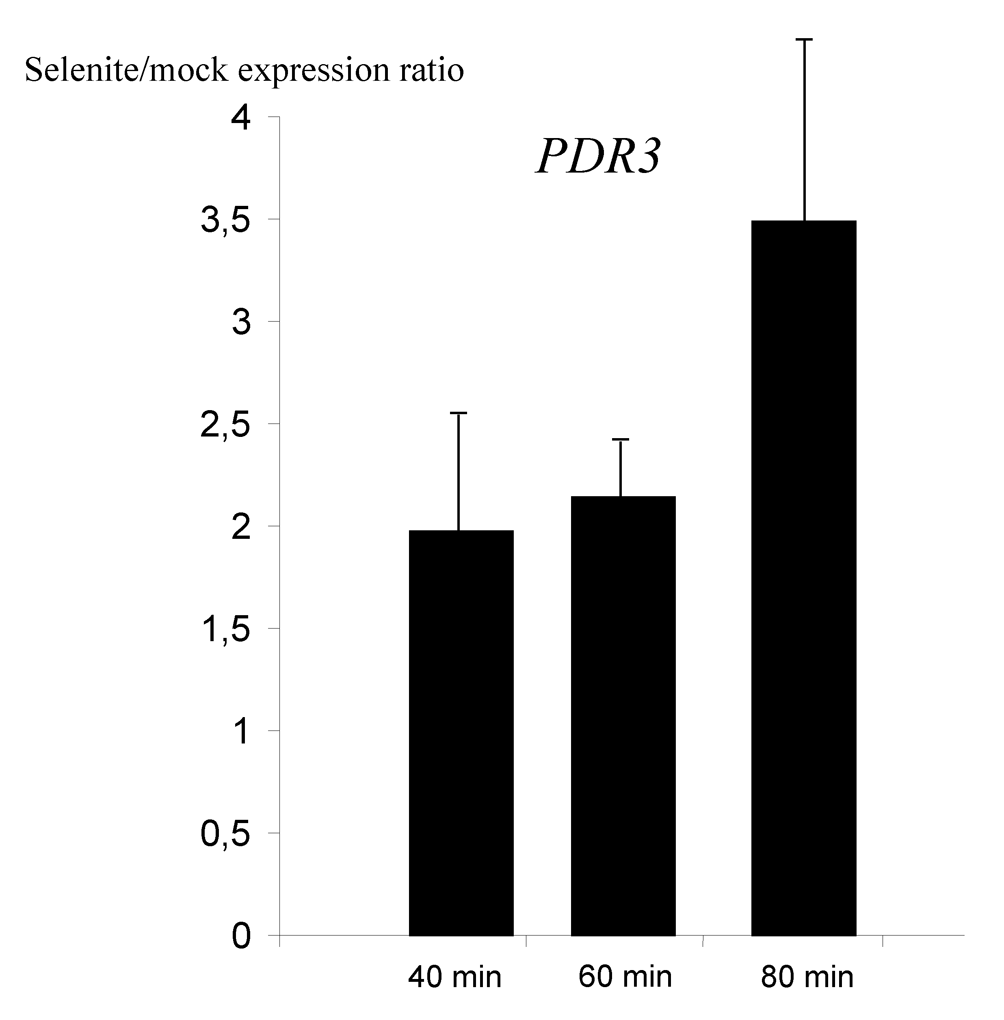

Supplement: Additional file 9 — Expression profile for PDR3 in response to selenite in wild-type yeast cells. Levels of PDR3 expression were quantified in wild-type cells, by real-time quantitative PCR. Expression values were normalized, using the gene encoding actin (ACT1, see methods). The values shown here are the ratios of normalized levels of PDR3 expression in the presence of selenite to normalized levels of expression of this gene in mock experiments. Each measurement was repeated three times, on independent samples. The standard errors are indicated. [file 1471-2164-9-333-S9.tiff]
